# Some sustained improvements in pneumonia case management four and five years following implementation of paediatric hospital guidelines in Lao PDR

**DOI:** 10.1038/s41598-017-10880-3

**Published:** 2017-09-06

**Authors:** Amy Gray, Louis Chhor, Saysanasongkham Sanyalack, Ruth Lim, Jana Lai, Keo Vilivong, Melinda Morpeth, Douangdao Soukaloun, Fiona Russell

**Affiliations:** 1 Centre for International Child Health, Department of Paediatrics, The University of Melbourne, The Royal Children’s Hospital, Parkville, Victoria Australia; 2 The Royal Children’s Hospital, Parkville, Victoria Australia; 3 Mahosot Hospital, Vientiane, Lao PDR; 4 University of Health Sciences, Vientiane, Lao PDR; 5 Pneumococcal Group, Murdoch Childrens Research Institute, The Royal Children’s Hospital, Parkville, Victoria Australia

## Abstract

In 2010, WHO paediatric hospital guidelines were implemented in Lao PDR, along with training workshops and feedback audits, achieving significant improvements in pneumonia case management when assessed one-year post-intervention. The sustainability of these improvements is hereby assessed, four and five years post-intervention. Medical records of children aged 1–59 months, diagnosed with pneumonia in 2010, 2011, 2014 and 2015 from a central Lao hospital were reviewed. Information relating to clinical steps in pneumonia case management was extracted and a scoring system applied based on the documentation of each clinical step, producing a pneumonia assessment score for each case. Comparisons of clinical steps and mean assessment score across study years were performed using Pearson’s chi-squared and t-tests, respectively. Of 231 pneumonia cases, the mean assessment scores in 2010, 2011, 2014 and 2015 were 57%, 96%, 69% and 69% respectively, showing a significant reduction from the immediate post-intervention period (2011) to 2015 (p < 0.01). Mean assessment score in 2014/2015 was significantly higher than in 2010 (p < 0.01). The high standards of pneumonia case management in 2011 were not observed in 2014/2015 in the absence of ongoing intervention but overall quality of care remained higher than pre-intervention levels, suggesting some degree of sustainability in the long-term.

## Introduction

Approximately one million children under five years of age died from pneumonia in 2015, of which almost 99% were from low- and middle-income countries (LMIC)^1^. Improving the quality of hospital care is an important strategy in reducing pneumonia mortality. One approach is the implementation of evidence-based clinical practice guidelines using various strategies such as training, dissemination, audit and supportive supervision^2^.

The ‘WHO Pocketbook of Hospital Care for Children’, released in 2005 and revised in 2013, is designed for use in hospitals with limited resources, and provides evidence-based guidelines for common childhood illnesses^3^. Many hospitals in LMIC introduced these guidelines but few countries have adapted them locally and incorporated them into everyday practice^4^. Even within national health systems where the Pocketbook has been introduced, there are differences in the uptake of the guidelines across regions and provinces, and across different stages of medical training^5^.

Few countries have assessed the impact of evidence-based guidelines on quality of hospital care for children with pneumonia. Of the available evidence from implementation efforts in Lao PDR (or Laos), Kenya and Malawi, implementation of guidelines in hospitals, in addition to specific training workshops and active clinical feedback has been shown to improve pneumonia case management^6, 7^, and reduce pneumonia-specific mortality^2^. However, evidence regarding the impact of guideline implementation on pneumonia case management beyond the first year following an intervention is scarce.

Improvements in the quality of pneumonia care is critical for Laos as it has one of the highest childhood mortality rates in South-East Asia, of which pneumonia is a major contributor^1^. In 2010, an intervention to implement a locally adapted version of the WHO Pocketbook led to significant improvements in pneumonia case management in three central Lao hospitals^7^. However, it is unknown whether these improvements have been sustained, in the absence of ongoing quality improvement strategies. In this study, the quality of care for childhood pneumonia was evaluated in one central hospital in Vientiane four and five years following the implementation of the Pocketbook.

## Methods

### Study site

The study hospital is a tertiary-level, major referral hospital in Vientiane, Laos, which admits >3000 children each year. Paediatric care is provided in a dedicated paediatric ward with intensive care capacity and on-site paediatricians, residents and nurses. Pulse oximeters, oxygen cylinders and oxygen delivery devices are available on the wards. Medications and antibiotics as recommended by guidelines are also available.

The WHO Pocketbook of Hospital Care for Children was implemented in three Lao hospitals in 2010 and has been described previously^7^. In brief, this multifaceted intervention included translation and adaptation of guidelines, introduction through interactive training workshops, audit and feedback on clinical practice and local consensus building with paediatricians.

### Study design

We conducted a retrospective cohort study. Data from the pre-intervention (2010) and one-year post-intervention (2011) periods were retrospectively collected in the previously published study^7^. This included data from three central hospitals. Only data from the relevant central hospital was extracted for use in the current study. For 2014 and 2015 data, medical records of eligible children were retrospectively reviewed using the same methods and selection strategy as used in the published 2010/2011 study^7^. Cases were selected from September for each year, and then for subsequent preceding months in reverse chronological order until the target number of cases had been reached for each year.

### Study population

Children aged 1–59 months and with a discharge diagnosis of pneumonia were eligible for inclusion. Medical records in which a diagnosis of pneumonia could not be confirmed based on the review of documented clinical features and available radiography results were included, but classified as ‘cough only’. Case records in which nursing notes were missing were excluded.

### Data collection

Clinical data related to pneumonia case management, as outlined below, and demographic data (including patient age, gender and length of stay) were abstracted from the medical records using the same data collection form from the previous study. Data was collected by the primary researcher (LC) and a senior Lao paediatric resident (SS) to ensure the interpretation of data was accurate. Access to data from 2010/2011 was granted permission from the primary researcher of the previous published study (AG).

### Clinical performance indicators

The study assessed care provided to children with pneumonia in the admission period. Clinical information was abstracted if recorded up to six hours from the documented time of first presentation to the hospital. The measured clinical indicators reflected key domains of pneumonia case management: assessment, diagnosis, treatment and monitoring. Each clinical indicator was assigned a score. (Table 1) Some indicators receiving greater weighting due to their relative importance in pneumonia case management (e.g. documentation of weight and oxygen saturation). Chest X-ray was not included as a key performance indicator as WHO guidelines do not mandate X-ray for non-severe pneumonia. In addition, in Lao PDR where patients pay for investigations which are performed clinical diagnosis of pneumonia without an X-ray is appropriate to limit out-of-pocket expenses.

**Table 1:** Pneumonia case management indicators measured and their individually assigned scores.

Clinical performance indicators (as documented in medical records)	Score
**Assessment**	
History	
Cough	0.5
Cough duration	0.5
Fever	0.5
Ability to drink	0.5
Treatment taken before presentation to hospital	0.5
Previous history of respiratory illness	0.5
Contact history with tuberculosis or person with chronic cough	0.5
Immunisation status	0.5
Examination	
Weight	1
Conscious state	0.5
Respiratory rate	0.5
Cyanosis	0.5
Oxygen saturation	1
Chest indrawing	0.5
Other signs of respiratory distress^a,b^	0.5
Auscultation findings	0.5
Diagnosis	
Pneumonia severity documented by clinician	1
Correct pneumonia severity classification^a^ (*based on available documented clinical information*)	1
Treatment	
Correct antibiotic prescribed for clinician-assigned severity^a^	1
Correct dosage of antibiotic^a^	1
Prescription of anti-cough or anti-histamine medication	−1
Monitoring	
Feeds and fluid intake documented	1
Adequate monitoring of vital signs^a,b^	1
**Total**	15

^a^Clinical indicator as defined by the WHO Pocketbook (1^st^ edition).

^b^Defined as grunting, nasal flaring, head nod or tracheal tug.

^c^Defined as at least twice in the first 24 hours of admission for non-severe pneumonia and at least four times in the first 24 hours for severe/very severe pneumonia.

Clinical indicators were dichotomous variables and scoring was allocated if a particular clinical step was documented in the medical records or if a particular clinical task was performed correctly as defined by the WHO Pocketbook guidelines^3^. Practice was assessed against the first edition of the WHO Pocketbook, which was updated in a second edition in 2013 and included changes to pneumonia classification and treatment. Although the new guidelines have since been incorporated into a revised Lao Pocketbook, this was not the case in 2014. Furthermore, the study authors reviewed the medical records aware of the potential influence of the new guidelines, but found no evidence of them in practice.

Scoring of cases as per Table 1, were performed by LC and in agreement with the primary researcher of the previous study (AG) to ensure consistency across the study years. In the treatment domain, ‘correct dosage of antibiotic prescribed’ was assigned if the antibiotic dose prescribed was within +/−10% of the WHO Pocketbook recommended dose/kg. Monitoring of a child’s vital signs were deemed adequate if vital signs were recorded at least twice in the first 24 hours of admission for non-severe pneumonia and at least four times in the first 24 hours for severe/very severe pneumonia^3^. Negative scoring was applied in instances where non-recommended practices of prescribing anti-cough or anti-histamine medications were documented.

Pneumonia case management is a pathway from (1) a complete assessment of essential clinical signs; (2) diagnosis and classification of severity; (3) antibiotic treatment according to severity and (4) monitoring (or discharge) according to severity classification^3^. Successful sequential completion of this care pathway, rather than individual steps, is another measure of cases management quality. Thus the proportion of cases in which the sequential steps in pneumonia management were documented correctly were also analysed using the current study data (2014 and 2015). This was compared with the previous study outcomes from all three hospitals as there was no significant difference in pneumonia case management between the three sites^7^.

### Sample size

A sample size of 40 cases in both 2014 and 2015 would provide 90% power to detect a 6% difference with 95% confidence in mean pneumonia case management scores between each year post-intervention. To perform secondary subgroup analyses, it was decided that for 2014 and 2015, sample sizes of 90 case records per year were to be collected.

### Analysis

Data collection forms were reviewed and any inconsistencies corrected by correlation with the medical record, before being entered into Epi-Data version 3.1^8^. Data were then exported, cleaned and analysed in STATA version 14^9^.

Continuous data for age and duration of stay were described by medians and interquartile ranges. Comparisons of age and duration of hospital stay across the study years were performed using Kruskal-Wallis tests. Categorical data for discharge outcome, pneumonia severity, gender and documentation of individual steps in pneumonia case management were described according to number and percentage of cases. Comparisons of percentages across study years were performed using Pearson’s chi-squared test. An alpha level of 0.05 was used to define statistical significance.

For each case record, the scores assigned for each clinical step were summed to produce an overall pneumonia case management score (totalling 15 points), expressed as a percentage of the maximum score where 15/15 points would be equal to 100%. Pneumonia case management scores were normally distributed for each study year (using Shapiro-Wilk tests). Data were summarised for each year using mean pneumonia case management score and 95% confidence intervals. Comparisons of scores between study years were performed using two-tailed t-tests, with an alpha level of 0.05. For all between year comparisons, results for 2014 and 2015 were combined, as there was no significant difference in mean case management score (p = 0.68).

### Ethical approval

Ethical approval was obtained from The Royal Children’s Hospital Human Research Ethics Committee (Reference number: 35212) and the Lao National Ethical Committee for Health Research. All methods were performed in accordance with the relevant guidelines and regulations. Written informed consent was obtained for data from 2014/2015 as data were collected primarily for a pneumonia aetiology study. As data from 2010/2011 were initially collected for audit and training purposes, written and verbal consent was obtained from the hospital director.

### Data availability statement

Datasets generated and analysed during this study are available from the corresponding author upon reasonable request.

## Results

The characteristics of the pneumonia cases included for each study year are shown in Table 2. There were no significant differences for age, gender, discharge outcome, pneumonia severity classification and duration of stay for included cases between years.

**Table 2:** Characteristics of pneumonia cases included in the study in a central hospital in Lao PDR in 2010, 2011, 2014–2015.

Characteristics	Pre-intervention^a^ (2010)	One-year post intervention (2011)	Four-years post intervention (2014)	Five-years post intervention (2015)	P-value
Cases, *n*	29	27	83	92	n/a
Age in months,					
Median (IQR)	11 (7–24)	10 (5–24)	9 (4–16)	15 (7–22)	0.21^b^
Male, *n* (%)	14 (48)	17 (63)	51 (61)	50 (54)	0.53^c^
Outcome, *n* (%)					
Well	27 (93)	26 (96)	78 (94)	87 (95)	0.98^c^
Unwell^d^	0 (0)	0 (0)	1 (1)	1 (1)	
Death	2 (7)	1 (4)	4 (5)	4 (4)	
Pneumonia severity^e^, *n* (%)					
Non-severe	7 (24)	0 (0)	12 (14)	16 (17)	0.13^c^
Severe	14 (48)	24 (89)	55 (66)	61 (66)	
Very severe	8 (28)	3 (10)	14 (17)	13 (14)	
Cough only^f^	0 (0)	0 (0)	2 (2)	2(2)	
Duration of stay in days,					
Median (IQR)	3 (2–6)	3.5 (3–4.5)	4 (3–5)	4(3–5)	0.96b

^a^Refers to multifaceted intervention to implement WHO Pocketbook guidelines in Lao PDR involving adaptation and translation of pocketbook, collaboration with local opinion leaders and clinical feedback audits^7^.

^b^P-value calculated using Kruskal Wallis test.

^c^P-value calculated using Pearson’s chi-squared test.

^d^Discharged home with signs of illness as requested by family.

^e^Pneumonia severity as defined by WHO Pocketbook (1^st^ edition).

^f^Classified when cough present but other signs of pneumonia absent on admission.

**Figure 1:**
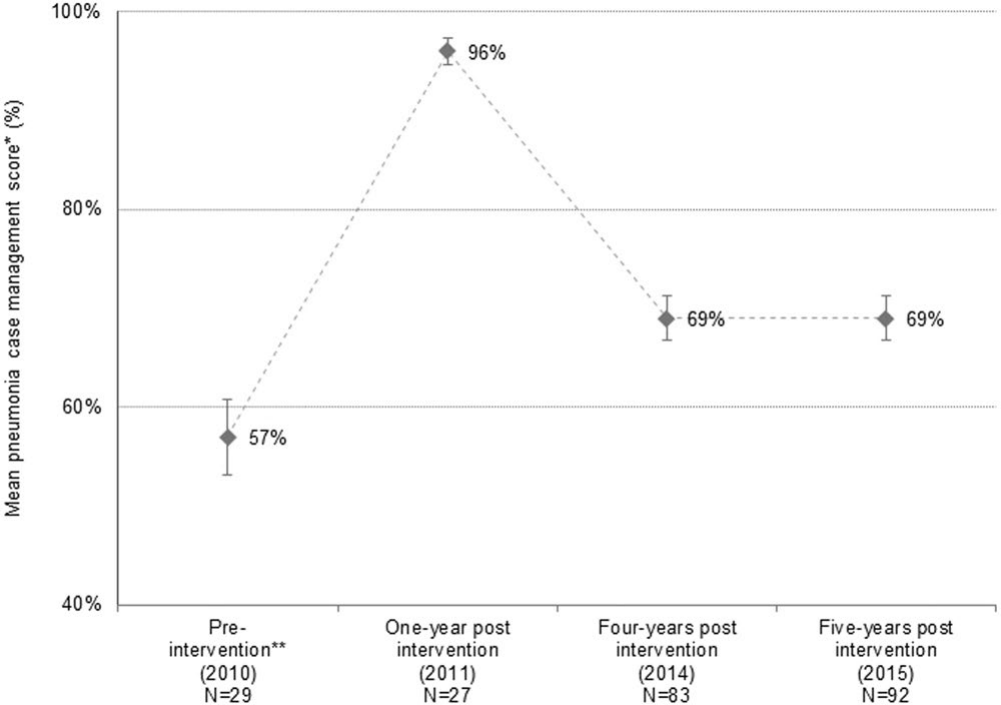
Pneumonia case management scores from pneumonia cases at the study hospital for each year relative to the intervention. *Scores presented as a percentage of the maximum score. Error bars show the 95% confidence interval of the mean. **Refers to a multifaceted intervention to implement WHO hospital guidelines in Lao PDR involving translation of guidelines, consensus building, local opinion leader engagement and clinical feedback audits.

The mean pneumonia case management scores for years relative to the intervention are shown in Fig. 1. At one-year post-intervention, the mean pneumonia case management score was 96%, increasing from 57% pre-intervention (p < 0.001). Mean pneumonia case management scores four and five years post-intervention were both 69%, significantly lower than one-year post-intervention scores (p < 0.001) but higher than the pre-intervention era (p < 0.001).

Table 3 shows the performance of case management steps for each study year. At four and five years post-intervention (2014/2015) there were no significant changes in the proportion of cases in which respiratory rate (p = 0.33) and chest in-drawing (p = 0.11) were documented, compared with one-year post intervention (2011). The proportion of cases in which ability to drink and oxygen saturation were documented was lower four to five years post-intervention, compared to one year post-intervention (p < 0.01). In the combined 2014/2015 group the proportion of cases in which a complete assessment for pneumonia severity was documented (ability to drink, respiratory rate, oxygen saturation and chest in-drawing) was the same as pre-intervention (p = 0.94).

**Table 3:** Documented management steps for pneumonia admissions at a central hospital in Lao PDR for years relative to intervention.

Performance and documentation of case management steps	Pre-intervention (2010) (n = 29)	One-year post intervention (2011) (n = 27)	Four-years post intervention (2014) (n = 83)	Five-years post intervention (2015) (n = 92)	p-value	
					Comparison^a^	Comparison^b^
**Assessment**						
Ability to drink, *n* (%)	20 (69)	27 (100)	34 (41)	50 (54)	<0.01	0.04
Respiratory rate, *n* (%)	22 (76)	27 (100)	81 (98)	88 (96)	0.33	<0.01
Oxygen saturation, *n* (%)	14 (48)	27 (100)	58 (70)	66 (72)	<0.01	0.02
Chest in-drawing, *n* (%)	26 (90)	27 (100)	78 (94)	82 (89)	0.11	0.76
Complete assessment for severity^c^, *n* (%)	10 (35)	27 (100)	25 (30)	34 (37)	<0.01	0.94
**Diagnosis**						
Correct pneumonia severity, *n* (%)	18 (62)	25 (93)	61 (73)	59 (64)	0.01	0.49
**Treatment**						
Correct antibiotic choice, *n* (%)	20 (69)	26 (96)	60 (72)	56 (60)	<0.01	0.78
Correct antibiotic dosing, *n* (%)	9 (31)	27 (100)	64 (77)	69 (75)	<0.01	<0.01
Anti-tussive/Anti-histamine use, *n* (%)	19 (66)	14 (52)	59(71)	64 (70)	0.06	0.61
**Monitoring**						
Adequate monitoring of vital signs, *n* (%)	7 (24)	27(100)	42 (51)	50 (54)	<0.01	<0.01

^a^Pearsons chi-squared test comparing one-year post intervention (2011) with combined data from 2014 and 2015.

^b^Pearsons chi-squared test comparing pre-intervention (2010) and combined data from four and five-years post-intervention (2014/2015).

^c^Defined as documentation of ability to drink, conscious state, respiratory rate, oxygen saturation and chest indrawing.

High levels of correct pneumonia severity classification at one-year post- intervention (93% (25/27)) were not sustained in 2014 and 2105 (73% (61/83) and 64% (59/92) respectively (p = 0.01). There was no significant difference in the proportion of cases in which correct pneumonia severity was documented in the combined 2014/2015 data compared to baseline (p = 0.49). For cases in 2014/2015 where the assigned pneumonia severity was incorrect, clinicians under-diagnosed (i.e. classified severity as non-severe pneumonia when documented clinical signs diagnostic of severe or very severe pneumonia were present) in 76% of cases.

In 2014 and 2015, the proportion of cases in which correct antibiotic choice (72% (60/83) and 60% (56/92) respectively) was documented was lower than one-year post-intervention, and comparable to baseline (69% (20/29), p = 0.78). Correct antibiotic dosing was also lower in 2014 and 2015 (77% (64/83) and 75% (69/92) respectively) compared to one year post-intervention, but remained significantly higher than pre-intervention levels (31% (9/29), p < 0.01). Anti-tussive/anti-histamine use was frequently documented in 2014/2015 cases and comparable to pre-intervention levels (66% (9/29), p = 0.61). In 2014/2015 macrolide antibiotics were prescribed in 13% (23/90) of cases, compared to 2/30 (6.7%) both pre-intervention and one year post-intervention. Oral cefixime was prescribed in only one case pre-intervention, no cases one year post-intervention, then in 4.3% (4/83) and 8.7% (8/92) of cases respectively in 2014 and 2015. Intravenous ceftriaxone was prescribed in 30% (9/30) of cases pre-intervention, no cases one year post-intervention, then in 11% (9/83) and 18% (17/92) cases in 2014 and 2015.

Adequate monitoring of the child’s vital signs occurred in all cases at one-year post-intervention. Performance of monitoring was not sustained four and five years post-intervention, occurring in 51% (p < 0.01) and 54% (p < 0.01) of cases in 2014 and 2015 respectively, but remained significantly higher when compared to the pre-intervention era (p < 0.01).

**Figure 2:**
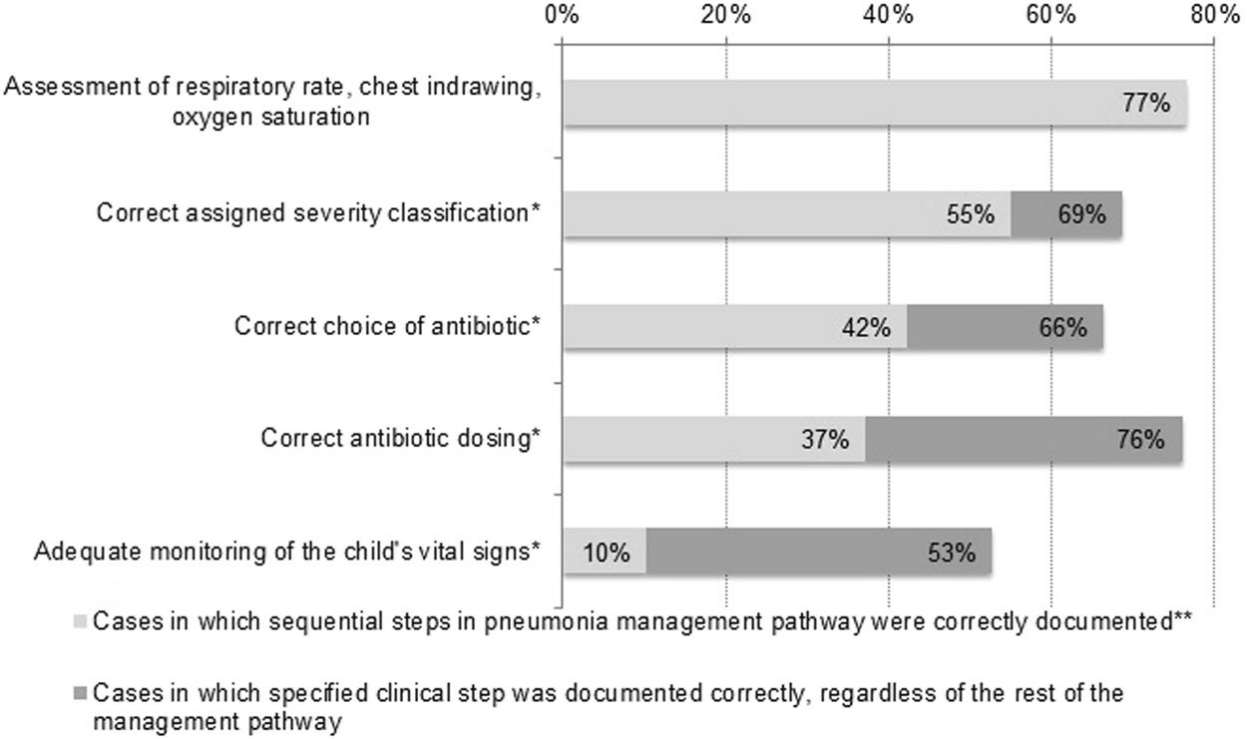
Percentage of pneumonia cases at the study hospital 4–5 years post intervention (2014/2015) in which performance of the sequential steps in the pneumonia case management pathway were documented correctly. *As defined by the WHO Pocketbook of Hospital Care for Children (1^st^ edition). **Values represent the percentage of cases in which sequential steps (ie the specified step and all preceding steps) in the management pathway were documented correctly.

Figure 2 shows the percentage of cases in which clinical steps are correctly documented sequentially from assessment to diagnosis, treatment and monitoring as recommended by the WHO Pocketbook guidelines^3^. The percentage of total cases in which the specified clinical step was documented is overlain for comparison. Only 10% of cases in 2014/2015 documented a complete and correct pneumonia case management pathway (i.e. documentation of respiratory rate, chest in-drawing and oxygen saturation, correct severity classification, correct antibiotic choice and dosing and adequate monitoring of vital signs). This was better than the pre-intervention period in which no patients had a correct pathway documented, but worse than one year post-intervention (62% in 2011 vs 10% in 2014/2015, p < 0.001).

## Discussion

The degree of improvement observed in pneumonia case management one-year following a guideline-based intervention in a central Lao hospital was not sustained in the absence of ongoing interventions four and five years post-intervention. However, the quality of pneumonia case management remained higher than the pre-intervention era. In this respect, there is some evidence of sustained improvement in quality of care but also evidence of deviation from care recommendations with time.

The changes in pneumonia case management scores overtime likely relate to the absence of an active intervention with decreasing awareness amongst clinicians of where their practice deviates from recommendations. There is also likely to be a tendency for practice to revert to “the mean” over time. In addition, staff turnover since the initial implementation efforts, in particular new resident staff and medical students who are most likely to document admission notes are likely to have not been exposed to the same degree of specific training around guideline use in general, or application to pneumonia more specifically. The re-emergence of broader antibiotic choices and non-recommended treatments such anti-tussives may reflect competing messages from sources such as drug marketing within hospitals. In 2014 and 2015, clinicians prescribed macrolide antibiotics in more than 10% of cases without obvious indications documented in the case records. This raises the possibility that staff may have been intending to treat atypical pneumonia caused by organisms such as *Mycoplasma pneumoniae* ^10^, as its contribution to childhood pneumonia has been described in neighbouring countries, including in children under five years of age^11, 12^. Rational macrolide use is not addressed in current WHO guidelines^3^, but may warrant future consideration given the observed practices.

It is important to note that correct antibiotic dosing and adequate monitoring of vital signs were more frequent at four and five years post-intervention compared to pre-intervention levels, while other practices such as antibiotic prescription returned to baseline levels. These may reflect practices that are more habitual, which are easier to change and sustain, as opposed to antibiotic choice which relies on clinician decision-making about the guideline recommendation, their perceptions of the suitability of the guideline for their patient or external influences on decision-making. The improvements found in adequate monitoring in the long term is encouraging as it is an aspect of care that requires interdisciplinary efforts, clarity of roles and expectations^13, 14^, and was an area of poor performance at baseline.

Future strategies should be directed at clinical aspects of care that were more poorly performed and quality improvement approaches to continually encourage clinicians to understand and close the gap between evidence-based recommendations and practice. A complete assessment for severity was seldom performed in 2014/2015 as danger signs, including the child’s ability to drink and the child’s conscious state, were not documented. While clinicians may be observing these signs but not documenting them, it can be argued that their documentation is also critical for ongoing monitoring of the child’s progress and determining their best management including use of fluids. Poor performance in documenting a child’s ability to drink has been described in many hospitals of LMIC, even where efforts have been made for guideline implementation^13, 15^. Furthermore under-diagnosis, the classification of true severe pneumonia as non-severe, was not uncommon and may reflect a lack of emphasis on the importance of key physical signs and their implications for severity of illness and mortality^16^.

As the direct impact of guideline implementation on hospital quality of care for childhood pneumonia in LMIC has not previously been evaluated for sustainability beyond one-year post-intervention, it is difficult to compare this study with others. Some guideline implementation studies in LMIC have reported on similar outcomes related to pneumonia case management and it would be interesting to know the sustainability of these outcomes in the long term for comparison^6, 13^. Though study designs, contexts and outcomes measured may differ, results relating to specific domains of pneumonia case management such as treatment may identify issues that are consistent across settings - such as the use of macrolides in this study.

In this study, clinical performance in pneumonia case management was assessed on the assumption that clinicians document the clinical steps they perform. This may underestimate actual performance but on the other hand may be justified by the importance of both performing and documenting the task for the purpose of patient management and monitoring change over time. Direct observation of the clinician in the assessment and management of the patient would provide a more objective assessment, but it is resource-intensive and time-consuming which made it unfeasible in our setting. Furthermore, this approach is likely to lead to observer bias in which clinicians may perform better when observed.

The outcome measures used in this study were chosen to reflect all aspects of pneumonia case management in the admission phase. Other quality assessment and guideline implementation studies have been more selective in their outcome measures relating to pneumonia care^6, 15, 17^, limiting the comparability of this study to others. The chosen process indicators do allow for identification of aspects of care that subsequently should become the focus for quality improvement and allow us to detect trends in clinical performance of assessment and management over time - which was the intention of this study. In contrast, Opondo *et al*. propose the use of a Paediatric Admission Quality of Care (PAQC) score, which unifies process indicators into domains of case management, better reflecting the algorithmic nature of guideline recommendations and allowing for more robust evaluation of clinical performance^18^.

Having an outcome measure that best captures guideline use as intended is important, but understanding why clinical tasks were poorly performed following guideline implementation may best be explained by qualitative insight into clinician perception of guidelines and in the instances that clinicians use them. Qualitative studies documenting barriers to the utility of the guidelines, clinical audits and training workshops in this setting will also help identify issues that should be addressed in future quality improvement strategies^19^. Nevertheless, this study has provided evidence that even once a large degree of change has been demonstrated, it cannot be assumed to be ongoing. Reinvigorating discussions of quality of care or ways to encourage practitioners to actively reflect on their practice is needed.

In conclusion, the major improvements in pneumonia case management observed one year after the intervention in a Lao hospital were not sustained to the same degree in the long term. But quality of care remained higher than pre-intervention levels providing reason to suggest that guidelines have a role in improving and sustaining these changes. Aspects of care which do not reflect guideline recommendations should become the focus for future quality improvement strategies at a hospital level. At a guideline level, due consideration of clinical concerns such as atypical pneumonia may need to be considered.

## References

[1] WHO. *Global Health Observatory data repository*, http://www.who.int/gho/en/ (2015). 10.1080/02763869.2019.1693231 32069199

[2] Enarson PM (2014). Reducing deaths from severe pneumonia in children in Malawi by improving delivery of pneumonia case management. PLoS ONE.

[3] WHO. *Pocket Book of Hospital Care for Children*. (World Health Organisation 2005).

[4] Li MY, Kelly J, Subhi R, Were W, Duke T (2013). Global use of the WHO pocket book of hospital care for children. Paediatrics & international Child Health.

[5] Li MY (2014). Implementation in Indonesia of the WHO Pocket Book of Hospital Care for Children. Paediatrics & international Child Health.

[6] Ayieko P (2011). A multifaceted intervention to implement guidelines and improve admission paediatric care in Kenyan district hospitals: a cluster randomised trial. PLoS Medicine / Public Library of Science.

[7] Gray AZ, Soukaloun D, Soumphonphakdy B, Duke T (2015). Implementing WHO hospital guidelines improves quality of paediatric care in central hospitals in Lao PDR. Tropical Medicine & International Health.

[8] EpiData (version 3.1). A comprehensive tool for validated entry and documentation of data (The EpiData Association, Odense, Denmark, 2003–2005).

[9] Stata Statistical Software: Release 14 (StataCorp LP, College Station, TX 2015).

[10] Biondi E, McCulloh R, Alverson B, Klein A, Dixon A (2014). Treatment of mycoplasma pneumonia: a systematic review. Pediatrics.

[11] Goyet S (2014). Etiologies and resistance profiles of bacterial community-acquired pneumonia in Cambodian and neighboring countries’ health care settings: a systematic review (1995 to 2012). PLoS ONE.

[12] Samransamruajkit R (2008). Prevalence of Mycoplasma and Chlamydia pneumonia in severe community-acquired pneumonia among hospitalized children in Thailand. Japanese Journal of Infectious Diseases.

[13] Irimu GW (2012). Performance of health workers in the management of seriously sick children at a Kenyan tertiary hospital: before and after a training intervention. PLoS ONE.

[14] Irimu GW (2014). Explaining the uptake of paediatric guidelines in a Kenyan tertiary hospital–mixed methods research. BMC Health Serv Res.

[15] Reyburn H (2008). Clinical assessment and treatment in paediatric wards in the north-east of the United Republic of Tanzania. Bull World Health Organ.

[16] Ayieko P, English M (2007). Case management of childhood pneumonia in developing countries. Pediatr Infect Dis J.

[17] Nolan T (2001). Quality of hospital care for seriously ill children in less-developed countries. Lancet.

[18] Opondo C, Allen E, Todd J, English M (2016). The Paediatric Admission Quality of Care (PAQC) score: designing a tool to measure the quality of early inpatient paediatric care in a low-income setting. Tropical Medicine & International Health.

[19] Gray, A. Z., Soukaloun, D. & Soumphonphakdy, B. A Qualitative Study of Provider Perceptions of Influences on Uptake of Pediatric Hospital Guidelines in Lao PDR. *American Journal of Tropical Medicine & Hygiene*, Accessed 13 June, 2017, doi:10.4269/ajtmh.16-1005 (2017). 10.4269/ajtmh.16-1005 PMC5544088 28722590

